# Supplementary material for: The Effect of Common Viral Inactivation Techniques on 16S rRNA Amplicon-Based Analysis of the Gut Microbiota
Source: Microorganisms. 2021 Aug 17;9(8):1755. doi: 10.3390/microorganisms9081755 (PMC8400488; doi:10.3390/microorganisms9081755)
Supplement: Supplementary file 1 [file microorganisms-09-01755-s001.zip › microorganisms-1318855-supplementary.pdf]

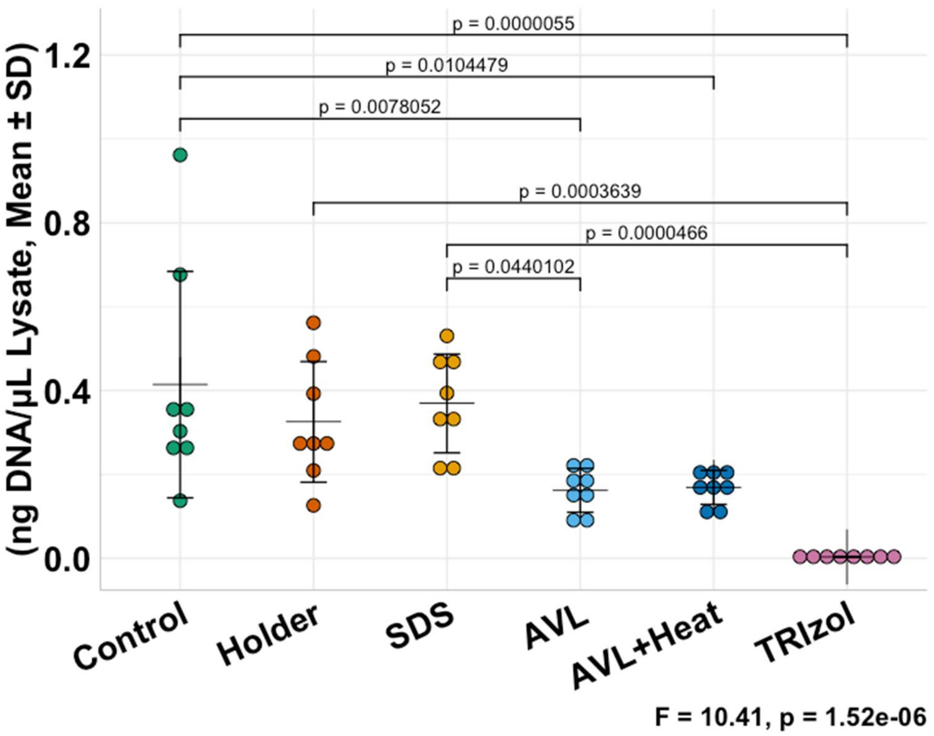

Supplementary Figure S1. Treatment-dependent differences in fecal lysate DNA concentration normalized to pellet mass. Control (*n* = 8), Holder (*n* = 16), SDS (*n* = 8), AVL (*n* = 8), AVL + Heat (*n* = 8), TRIzol (*n* = 8). Dots represent individual data points, bars represent mean ± SD, one-way ANOVA followed by Tukey HSD post-hoc test; *p* values for significant differences indicated.

Taxonomic Assignment	F	*p*	FDR	Tukey's HSD (*p* < 0.05)
d__Bacteria;p__Actinobacteriota;c__Coriobacteriia;o__Coriobacteriales;f__Eggerthellaceae;g__Enterorhabdus	9.20	4.93E-06	5.91E-4	Control-AVL<br>SDS-AVL<br>Holder-AVL + Heat<br>Holder-Control<br>SDS-Holder
d__Bacteria;p__Firmicutes;c__Bacilli;o__Erysipelotrichales;f__Erysipelotrichaceae;g__uncultured	7.88	2.67E-05	1.26E-3	Holder-AVL<br>Holder-AVL + Heat<br>Holder-Control<br>SDS-Holder
d__Bacteria;p__Firmicutes;c__Bacilli;o__Lactobacillales;f__Lactobacillaceae;g__Lactobacillus	7.75	3.14E-05	1.23E-3	Holder-AVL<br>Holder-AVL + Heat<br>Holder-Control<br>SDS-Holder
d__Bacteria;p__Firmicutes;c__Clostridia;o__Clostridia_vadinBB60_group;f__Clostridia_vadinBB60_group;g__Clostridia_vadinBB60_group	6.25	2.32E-4	6.97E-3	Control-AVL<br>SDS-AVL<br>Holder-Control<br>SDS-Holder
d__Bacteria;p__Patescibacteria;c__Saccharimonadia;o__Saccharimonadales;f__Saccharimonadaceae;g__Candidatus_Saccharimonas	6.04	3.11E-4	7.45E-3	Holder-AVL<br>Holder-AVL + Heat<br>Holder-Control

Supplementary Table S1. Summary of genera exhibiting significantly different abundances between inactivation methods, one-way ANOVA followed by Tukey HSD post hoc test, False Discover Rate (FDR), significant differences between groups ($p < 0.05$)

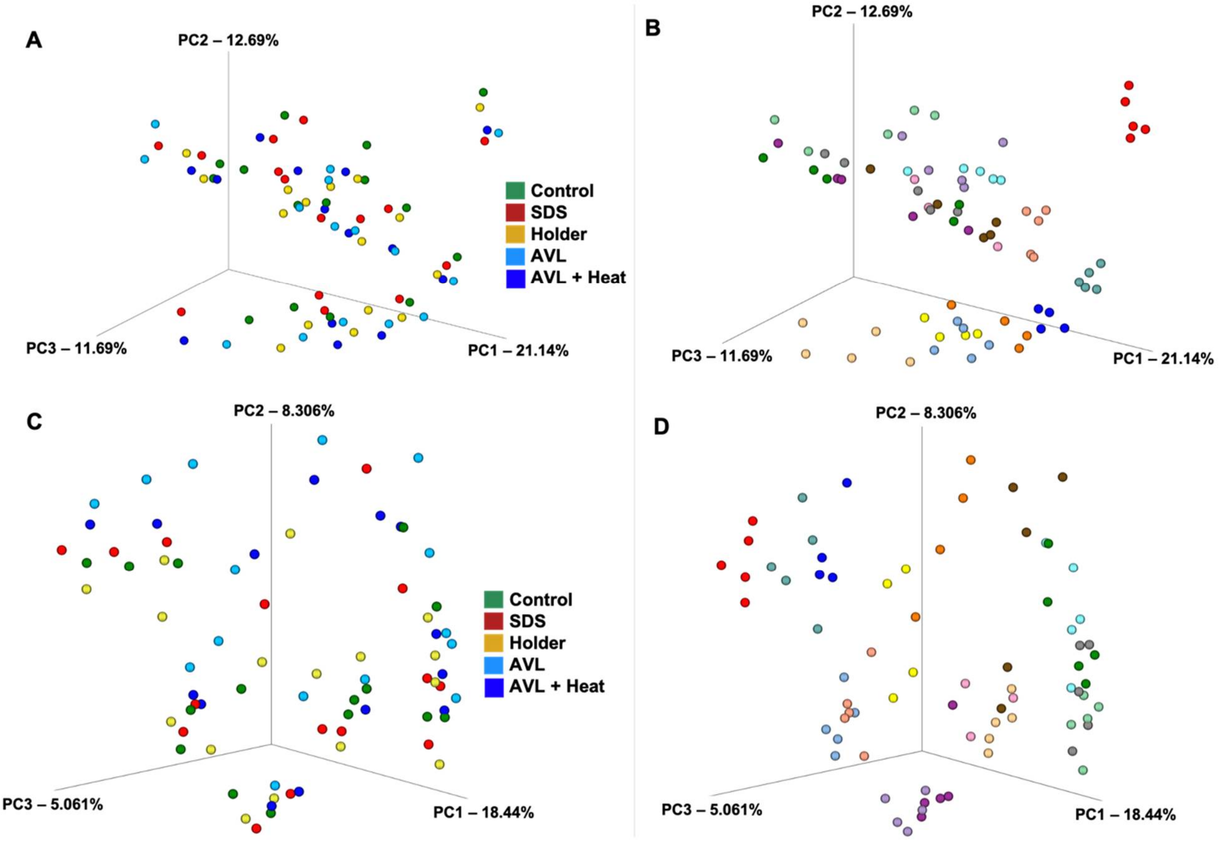

Supplementary Figure S2. Three-dimensional PCoA plots of samples labelled by inactivation method (A,C) and sample donor (B,D) using weighted (A-B) and unweighted (C-D) metrics. For samples labeled by donor, each color represents a unique donor.

Interactive three-dimensional PCoA plots can be accessed at https://github.com/ericsson-lab/viral-inactivation.git and are viewable at view.qiime2.org.
